# Altered sperm tsRNAs in aged male contribute to anxiety‐like behavior in offspring

**DOI:** 10.1111/acel.13466

**Published:** 2021-08-27

**Authors:** Yi Guo, Dandan Bai, Wenqiang Liu, Yingdong Liu, Yalin Zhang, Xiaochen Kou, Jiayu Chen, Hong Wang, Xiaoming Teng, Ji Zuo, Shaorong Gao

**Affiliations:** ^1^ Department of Cellular and Genetic Medicine School of Basic Medical Sciences Fudan University Shanghai China; ^2^ Clinical and Translational Research Center of Shanghai First Maternity and Infant Hospital Shanghai Key Laboratory of Signaling and Disease Research Frontier Science Center for Stem Cell Research School of Life Sciences and Technology Tongji University Shanghai China

**Keywords:** Advanced paternal age, anxiety, offspring, pre‐implantation embryo, sperm, transfer RNA‐derived small RNA

## Abstract

Parental age at first pregnancy is increasing worldwide. The offspring of aged father has been associated with higher risk of several neuropsychiatric disorders, such as schizophrenia and autism, but the underlying mechanism remains elusive. Here we report that advanced paternal age in mice alters the profile of transfer RNA‐derived small RNAs (tsRNAs). Injection of sperm tsRNAs from aged male mice into zygotes induced anxiety‐like behaviors in F1 males. RNA sequencing of the cerebral cortex and hippocampus of those F1 male mice altered the gene expression of dopaminergic synapse and neurotrophin. tsRNAs from aged male mice injection also altered the neuropsychiatry‐related gene expression in two‐cell and blastocyst stage embryos. More importantly, the sperm tsRNA profile changes significantly during aging in human. The up‐regulated sperm tsRNA target genes were involved in neurogenesis and nervous system development. These results suggest that aging‐related changes of sperm tsRNA may contribute to the intergenerational transmission of behavioral traits.

## Graphical abstract

Advanced paternal age in mice and human alters the profile of transfer RNA‐derived small RNAs (tsRNAs). Injection of sperm tsRNA from aged male mice into zygotes induced anxiety‐like behaviors in F1 males and also altered the neuropsychiatry‐related gene expression in two‐cell and blastocyst embryos. These results suggest that aging‐related changes of sperm tsRNA may contribute to the intergenerational transmission of behavioral traits.

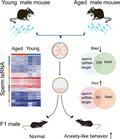

## 1 INTRODUCTION

Fathers of advanced age are increasingly common. Advanced paternal age has been associated with increased risk of stillbirth and various problems in offspring, including musculo‐skeletal syndromes, cleft palate, acute lymphoblastic leukemia, retinoblastoma, neurodevelopmental disorders on the autism spectrum, schizophrenia, and related behavioral traits (Nybo Andersen & Urhoj, 2017). Previous studies suggested that neuropsychiatric disorders in the offspring of aged fathers may be related to the high rate of *de novo* mutations in the sperm genome (Cioppi et al., 2019; Kong et al., 2012). Spermatogenesis begins at puberty and spermatogonial stem cells divide continuously throughout the male lifespan. Sperm mutation load accumulates during multiple cell divisions as a result of replicative errors. Another contributing factor may be epigenetic modifications in sperm, which correlate with the incidence of various diseases in offspring (Krug et al., 2020; Milekic et al., 2015; Su & Patti, 2019). For example, alterations in methylation of paternal sperm DNA have been associated with neuropsychiatric disorders in offspring (Jenkins et al., 2014; Milekic et al., 2015). In fact, sperm microRNAs (miRNAs) have been proposed to mediate the intergenerational transmission of genetic diseases such as anxiety, depression, and metabolic disorders (Dupont et al., 2019; Rodgers et al., 2015; Short et al., 2016; Wang et al., 2021). Transfer RNA‐derived small RNAs (tsRNAs) play a critical role in multiple processes, including maintenance of mRNA stability (Goodarzi et al., 2015; Oberbauer & Schaefer, 2018), gene silencing (Garcia‐Silva et al., 2012), reverse transcription (Ruggero et al., 2014), and gene regulation (Kuscu et al., 2018). In mice and humans, sperm tsRNAs appear to transmit metabolic traits from fathers to offspring (Chen et al., 2016; Donkin et al., 2016; Sharma et al., 2016). In mice, sperm tsRNAs regulate various genes in the pre‐implantation embryo (Sharma et al., 2016). Levels of sperm tsRNAs can be modified through the diet, and injecting sperm tsRNAs from males on a high‐fat diet into normal zygotes alters expression of metabolic genes, leading to metabolic disorders in the F1 offspring (Chen et al., 2016). In addition, Short et al. demonstrated that exercise altered both sperm miRNA and sperm tsRNA in mouse, which play an important role in the transgenerational modification of male offspring conditioned fear and anxiety (Short et al., 2017).

In order to explore whether sperm tsRNAs may contribute to risk of neuropsychiatric disorders in offspring, we conducted a study aimed to compare the behavioral traits of F1 progeny grown from zygotes injected with sperm tsRNAs from young or aged fathers. We show that the sperm tsRNA profiles are changed associated with advanced male age and conferred paternally acquired neuropsychiatric disorders to F1 offspring. Injection of sperm tsRNAs from aged male mice into zygotes induced anxiety‐like behaviors in F1 males and altered expression of nerve development genes in embryos and nerve tissue in F1 males. More importantly, further study in human shows that the changes of sperm tsRNAs in aged men are related to neurogenesis and nervous system development according to the gene ontology analysis.

## 2 RESULTS

### 2.1 Paternal aging is associated with increased anxiety‐like behavior in F1 males via sperm RNAs

**FIGURE 1:**
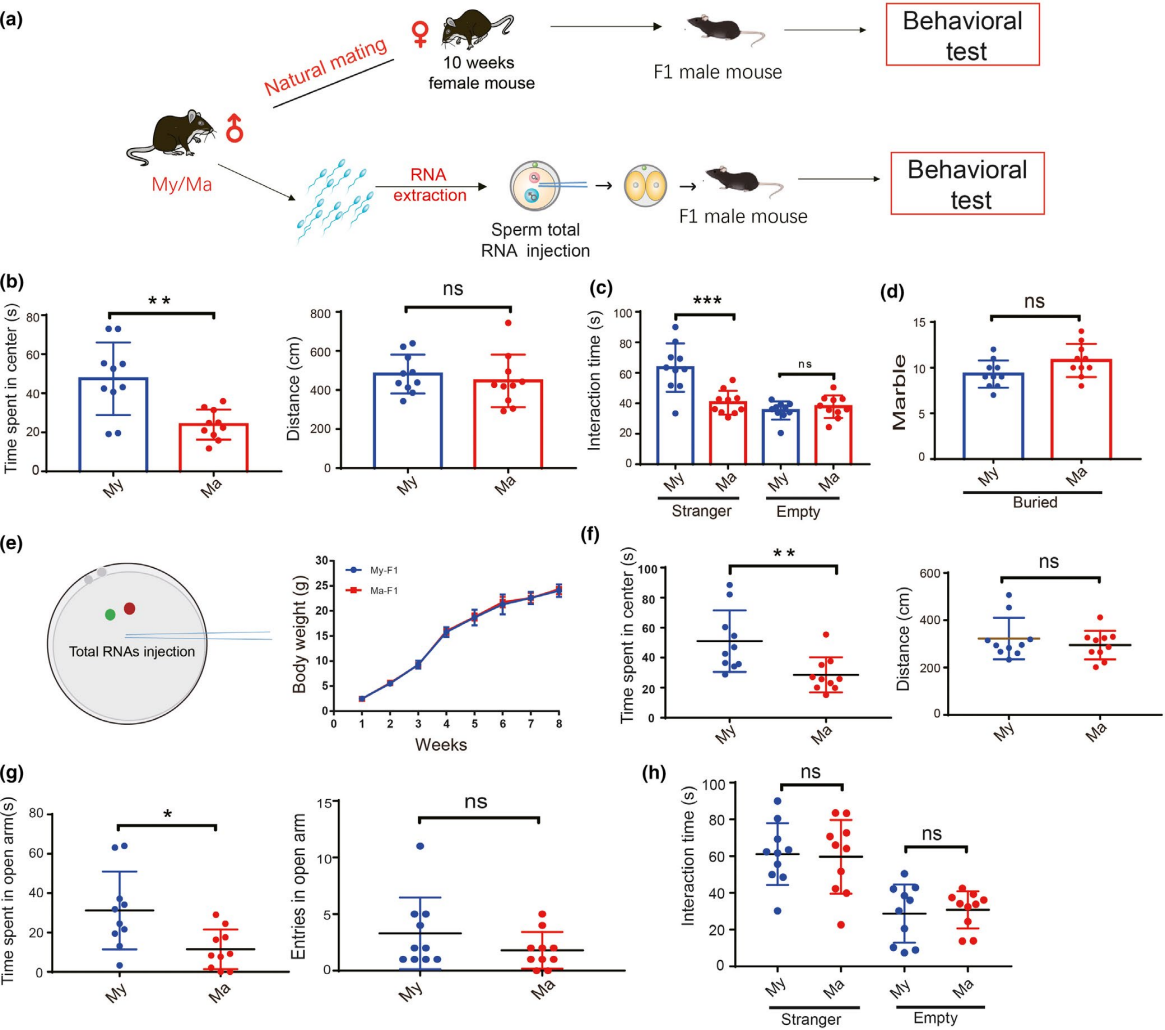
Behavioral traits in F1 mice arising from natural mating or from zygotes injected with total sperm RNAs from aged or young mice. (a) Schematic of the potential role of sperm RNAs in mediating hereditary transmission of anxiety‐like behavior to F1 male mice. (b‐d) Behavioral tests for F1 male offspring (*n* = 10 for each group) obtained by naturally mating female mice 10 weeks old with male mice 14–18 months old (aged male mice, Ma) or male mice 3–4 months old (young male mice, My). (b) Evaluation of anxiety‐like behavior in the open field test. (c) Evaluation of social ability in the three‐chambered social interaction test. (d) Evaluation of stereotypic behavior in the marble‐burying test. (e‐h) F1 male mice (*n* = 10 for each group) arising from zygotes injected with total sperm RNAs from Ma or My groups. (e) Scheme of injection of sperm total RNA into normal zygotes and growth curves of the resulting F1 male offspring. (f) Evaluation of anxiety‐like behavior in the open field test. (g) Evaluation of anxiety‐like behavior in the elevated plus‐maze test. (h) Evaluation of social ability in the three‐chambered social interaction test. *p < 0.05; **p < 0.01; ns, not significant

To examine the influence of aged males on offspring mental behavior, the female mice of 10 weeks old were mated with male mice 14–18 months old (hereafter “aged male mice” or Ma) or male mice 3–4 months old (hereafter “young male mice” or My). Then we conducted behavioral testing on the F1 males (*n* = 10 for each group) (Figure 1a).

In the open field test, time spent in the central area was significantly different between F1 males from Ma mated group and My mated group (24.0 ± 2.4 vs 47.4 ± 5.9s, *p*=0.002), while total travelled distance did not differ significantly between the two groups (Figure 1b). In the three‐chambered social interaction test, the time spent in the stranger chamber was shorter for F1 mice from aged fathers than for F1 mice from young fathers (40.4 ± 2.5 vs 63.4 ± 5.0s, *p* < 0.001; Figure 1c), while the time spent in the empty chamber did not differ significantly between the two groups. Similarly, the two groups did not differ significantly in the number of beads buried in the marble‐burying test (Figure 1d).

Next, we extracted total sperm RNAs from aged or young male mice and injected it into zygotes in order to obtain F1 males (Figure 1a). The two groups of F1 offspring did not differ significantly in their growth rate (Figure 1e). In the open field test, time spent in the central area was lower in the Ma total RNA injection group compared with the My total RNA injection group (28.6 ± 3.7s vs.51.0 ± 6.5s *p* = 0.007), while total distance travelled did not differ significantly between the two groups (Figure 1f). In the elevated plus‐maze test, the time spent on the open arm was significantly shorter in the Ma total RNA injection group (11.5 ± 3.2s vs. 31.2 ± 6.3s, *p* = 0.011), while the number of entries into the open arm did not differ significantly between the two groups (Figure 1g). In the three‐chambered social interaction test, the two groups did not show significant differences in the time spent in the stranger or empty chambers (Figure 1h). Overall, F1 male offspring from injection of Ma sperm total RNA reproduced the similar anxiety‐like behavioral alterations as male offspring born to aged male mice by natural mating.

### 2.2 Aging changed sperm tsRNA profile

To further investigate how sperm RNA conferred anxiety‐like behavior in F1 males, we examined the sperm sncRNA profiles of the Ma and My males by small RNA‐seq.

**FIGURE 2:**
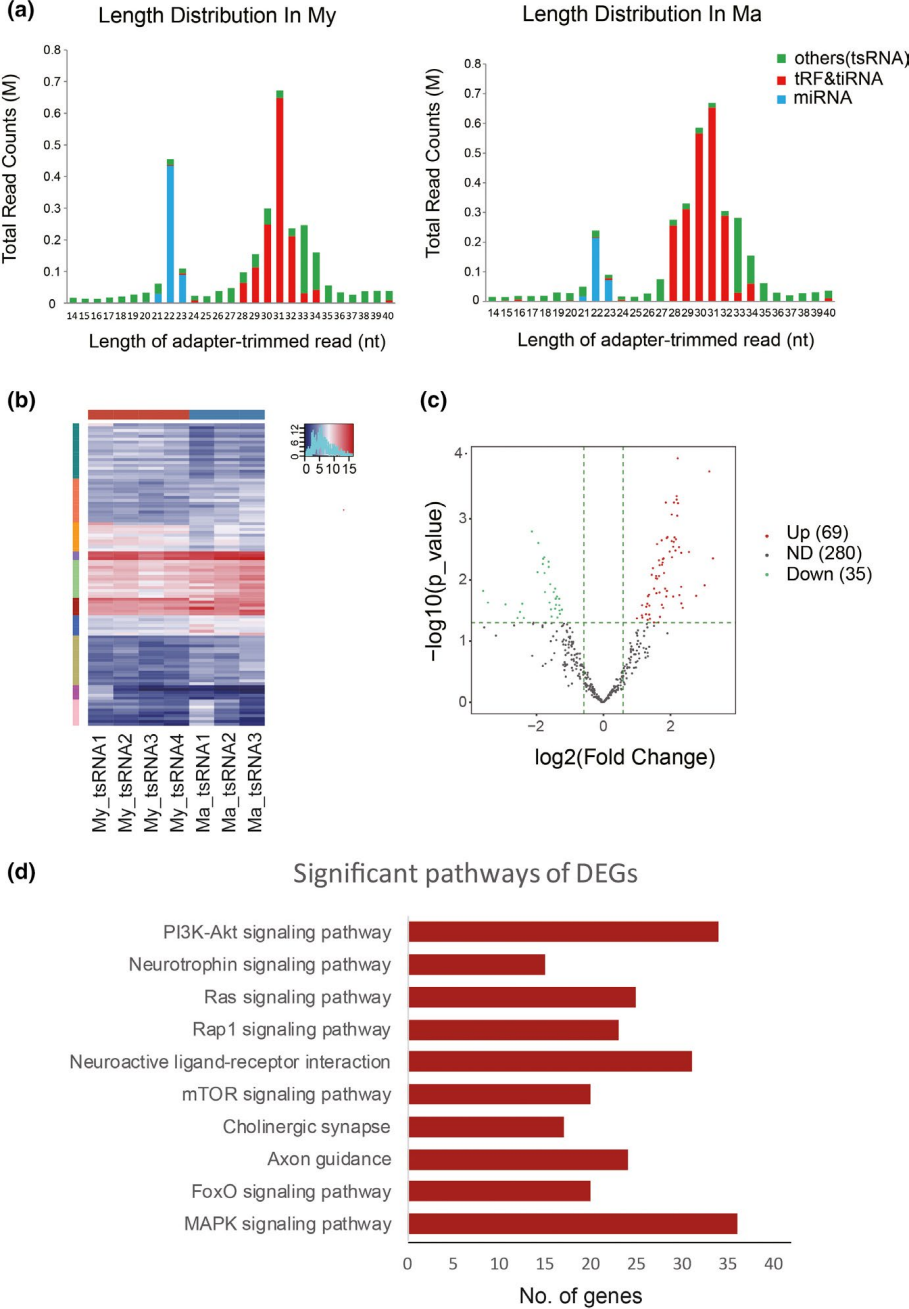
Aging alters the sperm tsRNA profile in mice. Sperm tsRNAs from aged male mice (Ma) and young male mice (My) were detected using Small RNA‐seq. (a) Distribution of read lengths. (b) Heat map of sperm tRNA‐derived fragments (tRFs and tiRNAs) differentially expressed between the Ma group and My group. (c) Volcano plot of sperm tRFs and tiRNAs differentially expressed between the Ma group and My group. (d) KEGG pathway analysis of differentially expressed tRFs and tiRNAs that were up‐regulated in the Ma group. Data are from *n* = 3 independent samples for the Ma group and *n* = 4 for the My group

Small RNA‐seq of sperm showed enrichment of miRNAs of 21–23 nucleotides and tsRNAs of 28–40 nucleotides in aged and young mice (Figure 2a). Among the 54,000 tsRNAs detected, 1,202 were up‐regulated and 408 were down‐regulated in the Ma males (Figure S1a, b). According to the tsRNA nomenclature rules reported by Kumar et al. (Kumar et al., 2016), a total of 384 tRNA‐derived fragments (tRFs) and tRNA‐derived stress‐induced RNAs (tiRNAs) were detected, of which 5′‐tsRNAs (tRF‐5c and tiRNA‐5) subtype accounted for a significant proportion in both groups (Figure S2a, b). 69 tRF and tiRNAs were up‐regulated and 35 down‐regulated in the Ma males (Figure 2b, c). Based on the seed sequence, the 69 up‐regulated tRF and tiRNAs could be grouped into 22 types (Table S1). Kyoto Encyclopedia of Genes and Genomes (KEGG) analysis showed that 22 up‐regulated tRF and tiRNA target genes were involved in nervous system‐related signaling pathways, including neurotrophin signaling, cholinergic synapses, and axon guidance (Figure 2d). In summary, the above results illustrated that sperm tsRNAs were indeed differentially expressed in aged mice, and these changes were involved in neuropsychiatric disorders pathway.

### 2.3 Sperm tsRNA from aged mice conferred F1 male behavior changes

**FIGURE 3:**
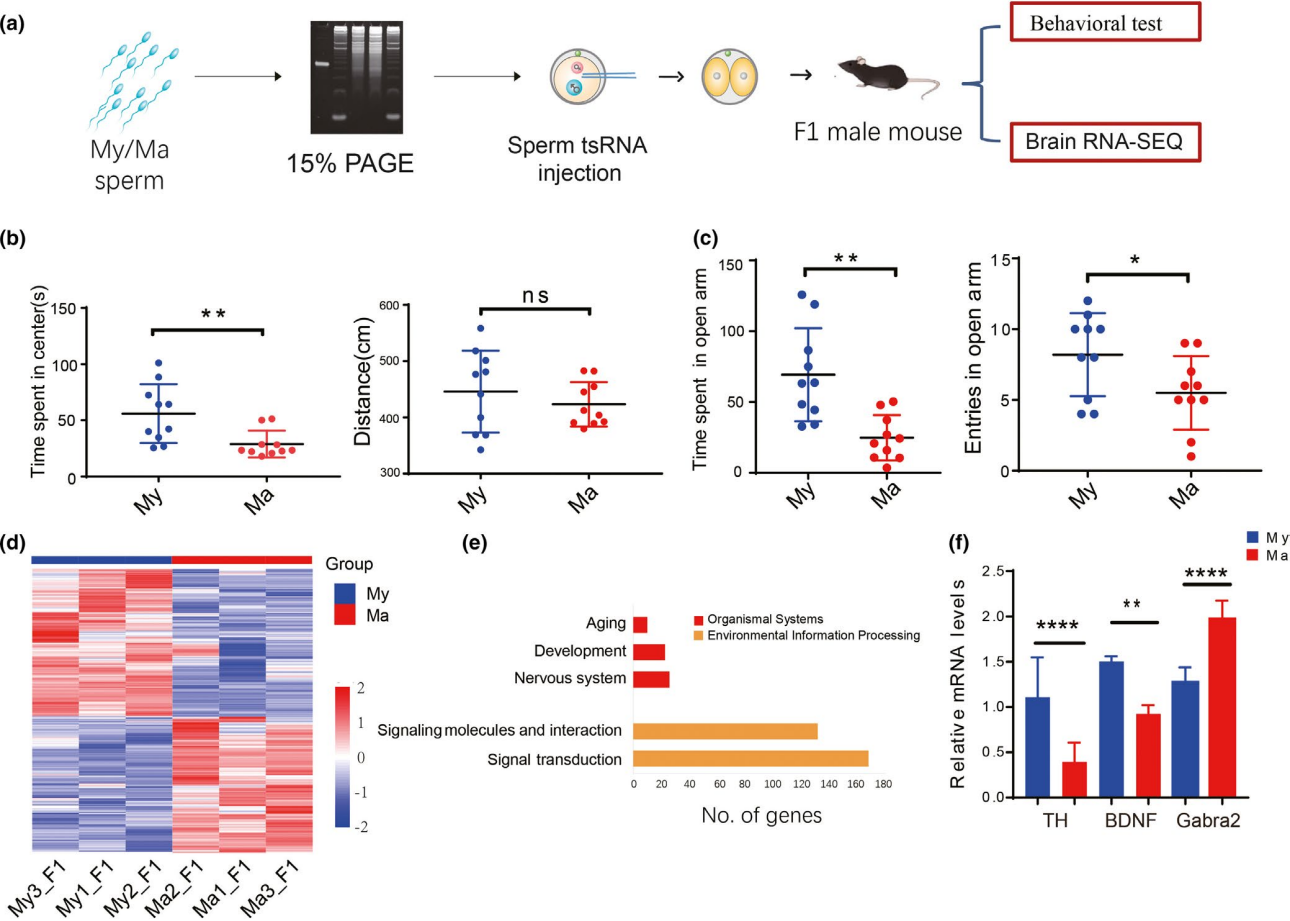
Sperm tsRNA from aged mice is associated with anxiety‐like behavior in F1 male mice. (a) Schematic of the procedure for evaluating the potential role of sperm tsRNA in hereditary transmission of anxiety‐like behavior to F1 male mice. F1 male offspring were obtained from zygotes that had been injected with sperm tsRNAs from aged male mice (Ma) or young male mice (My) (*n* = 10 mice for each group). (b) Evaluation of anxiety‐like behavior in the open field test. (c) Evaluation of anxiety‐like behavior in the elevated plus‐maze test. (d) Heat map of genes differentially expressed between the Ma group and My group in the cerebral cortex and hippocampal tissue. (e) KEGG enrichment analysis of differentially expressed genes in the cerebral cortex and hippocampal tissue. (f) Validation of some differentially expressed genes based on quantitative PCR. *p < 0.05; **p < 0.01; ***p < 0.001; ****p < 0.0001, ns: not significant

To investigate whether sperm tsRNAs are response for transgenerational of acquired traits, we injected zygotes with the “28–40 nt” fraction of sperm RNA, predominantly tsRNAs, from aged or young mice (Figure 3a; Figure S3a). The resulting groups of F1 males did not differ significantly in growth rate (Figure S3b).

In the open field test, time spent in the central area was significantly different between Ma tsRNA injection group and My tsRNA injection group (28.9 ± 3.8s vs 56.3± 8.3s, *p* = 0.008), while total distance travelled did not differ significantly between the two groups (Figure 3b). In the elevated plus‐maze test, the time spent on the open arm was significantly shorter in the Ma tsRNA injection group (24.9 ± 5.1 vs. 69.3 ± 10.4s, *p* = 0.001). The number of entries into the open arm was lower in the Ma tsRNA injection group (5.5 ± 0.8 vs. 8.2 ± 0.9, *p* = 0.04) (Figure 3c).

To further investigate the effects of sperm tsRNAs injection on nervous system of F1 males, we performed RNA‐seq of the cerebral cortex and hippocampal tissues of offspring. The result showed that 259 genes were down‐regulated and 237 genes were up‐regulated in the Ma tsRNA injection group compared to the My tsRNA injection group (Figure 3d; Figure S3c). Differentially expressed genes (DEGs) were related to aging, development, the nervous system, and neurodevelopmental pathways, according to KEGG analysis (Figure 3e). DEGs included the genes encoding tyrosine hydroxylase (TH), tryptophan hydroxylase 2 (TPH2), subunit rho 2 (Gabrr2) of the gamma‐aminobutyric acid C receptor, subunits alpha 6 (Gabra6) and alpha 2 (Gabra2) of the gamma‐aminobutyric acid A receptor, brain‐derived neurotrophic factor (BDNF), and nerve growth factor receptor (NGFR). Levels of mRNAs encoding BDNF, TH, and Gabra2 were determined by quantitative polymerase chain reaction (qPCR), which gave results consistent with RNA‐seq (Figure 3f). These data strongly suggested that F1 male offspring from injection of Ma sperm tsRNA gave rise to anxiety‐like behaviors, which might be due to the change of sperm tsRNA.

### 2.4 Effects of sperm tsRNA from aged mice on the pre‐implantation embryo

**FIGURE 4:**
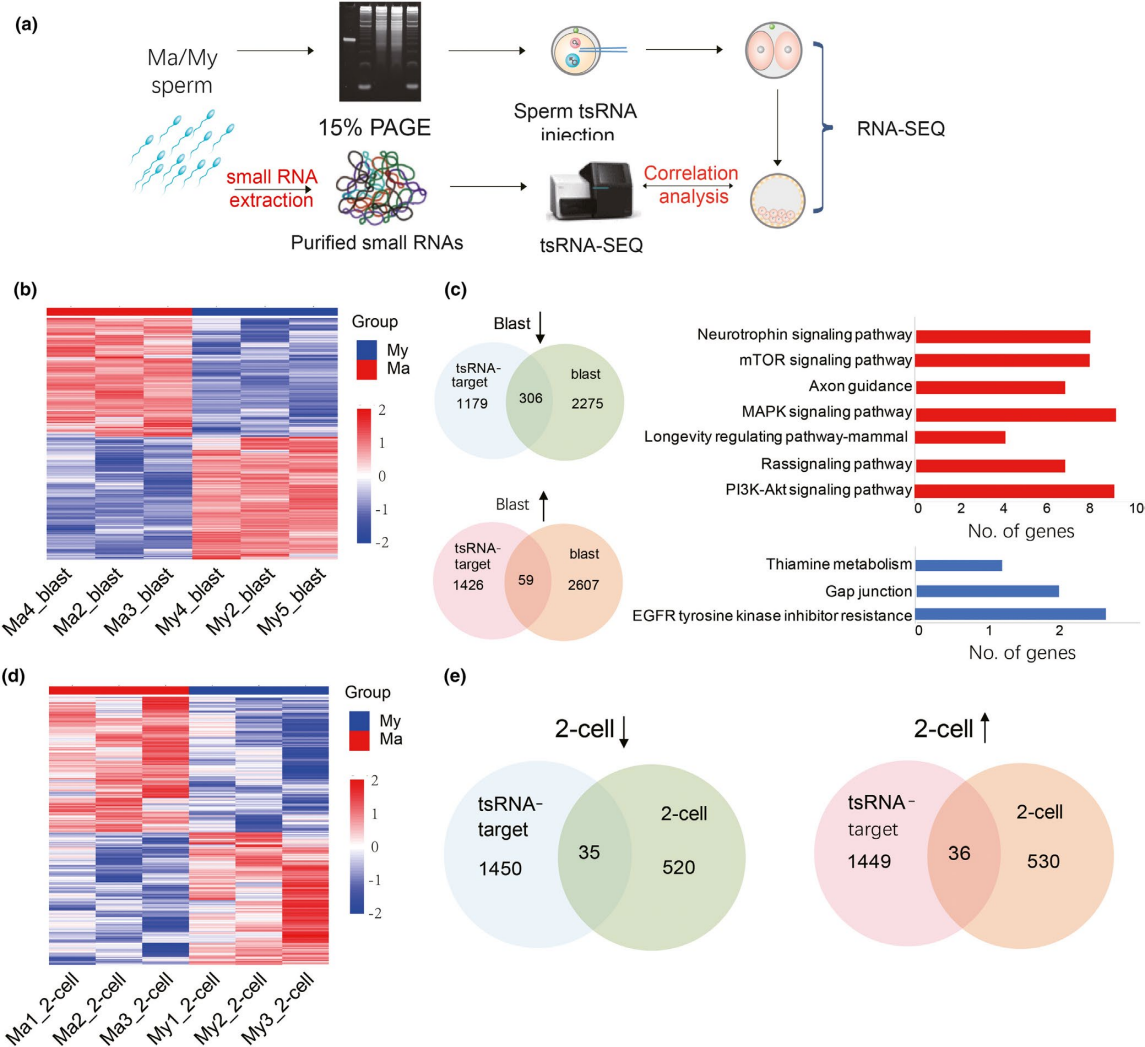
Sperm tsRNAs from aged mice dysregulate gene expression in pre‐implantation embryos. (a) Schematic of how gene expression was analyzed in pre‐implantation embryos. (b) Heat map of DEGs from blastocysts from the aged mouse (Ma) group and the young mouse (My) group. (c) Venn diagrams showing the intersection of genes targeted by differentially expressed tsRNAs and differentially expressed genes in blastocysts. KEGG pathway analysis of genes up‐ or down‐regulated in the Ma group relative to the My group. (d) Hierarchical cluster analysis (heat map) of results from two‐cell embryos from the Ma group and My group. (e) Venn diagrams showing the intersection of genes targeted by differentially expressed tsRNAs and differentially expressed genes in two‐cell embryos. Data are from *n* = 3 independent samples for each group

To verify if the injection sperm tsRNAs altered gene expression of pre‐implantation embryos, we injected with sperm tsRNAs from aged or young mice into the zygote respectively and performed RNA‐seq at two‐cell and blastocyst stage (Figure 4a). RNA‐seq of blastocysts identified 2581 down‐regulated genes and 2666 up‐regulated genes in the Ma group relative to the My group (Figure 4b). Several down‐regulated genes were related to neurological diseases and nerve signal transduction, based on KEGG analysis (Figure S4a). In the blastocyst of Ma tsRNA injection group, 306 down‐regulated genes overlapped with target genes of differentially expressed tsRNAs, and many of them were related to neural signaling pathways (Figure 4c). RNA‐seq of two‐cell embryos showed that 555 genes were down‐regulated and 566 genes were up‐regulated in the Ma group relative to the My group (Figure 4d). Several down‐regulated genes were related to neurotrophin signaling, cholinergic synapses, and other neural signaling pathways (Figure S4b). A total of 35 down‐regulated genes and 36 up‐regulated genes of Ma tsRNA injection group overlapped between the target genes of the differentially expressed tsRNAs and the DEGs in two‐cell embryos (Figure 4e). The results suggested that the changes of aging sperm tsRNAs might cause profound downstream effects during early embryonic development, which was related with neuropsychiatric disorders.

### 2.5 Aging alters the sperm tsRNA profile in human

**FIGURE 5:**
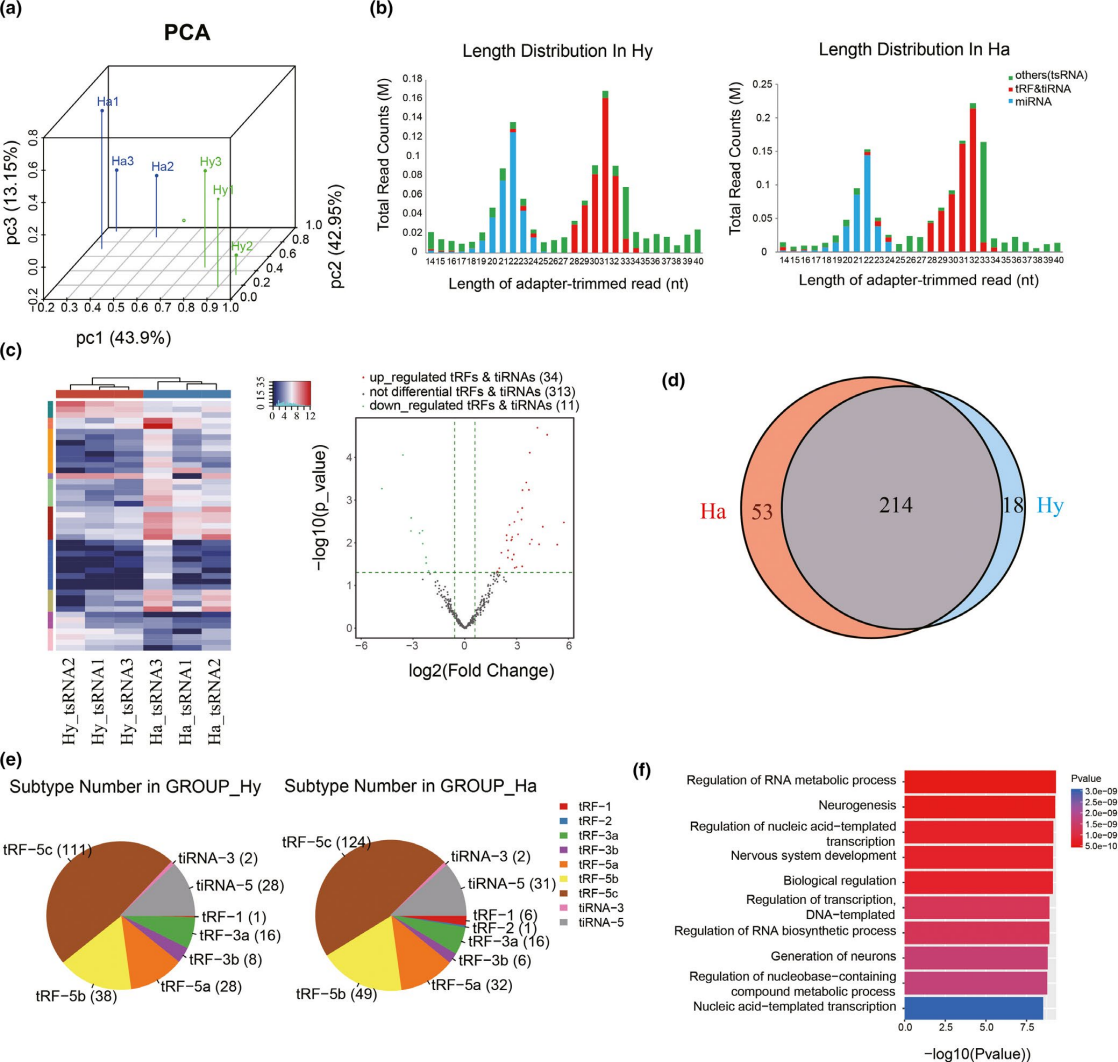
Aging alters the sperm tsRNA profile in men. Sperm tsRNAs from aged male human (Ha) and young male human (Hy) were detected using Small RNA‐seq. (a) Principal Component Analysis (PCA) on RNA‐Seq data from the Ma group and My group. (b) Distribution of read lengths. (c) Heat map and volcano plot of sperm tRNA‐derived fragments (tRFs and tiRNAs) differentially expressed between the Ma group and My group. (d)Venn diagram based on number of specifically expressed tRFs and tiRNAs. (e) Distribution of tRF and tiRNA subtypes. The numbers in brackets indicate the number of tRF or tiRNA in that subtypes. (f) Enrichment of gene ontology (GO) terms among tRFs and tiRNAs up‐regulated in Ha relative to Hy. Data are from *n* = 3 independent samples for each group

To further verify whether aging alters the sperm tsRNA profile in human, we examined the sperm sncRNA profiles of the young and aged man by small RNA‐seq. In principal component analysis, aged male human (Ha) and young male human (Hy) showed clear differences in tRFs and tiRNAs expression (Figure 5a). Small RNA‐seq of human sperm showed enrichment of tsRNAs at 28–34 nucleotides in Ha and Hy group (Figure 5b). Among the 358 tRFs and tiRNAs detected, 34 were up‐regulated and 11 were down‐regulated in the Ha group (Figure 5c). Of the 358 species, 214 were expressed in both groups (the values of counts per million of total aligned reads (CPM) were more than 20 in both two groups), 53 specific in the Ha group, and 18 specific in the Hy group (the specific represent the CPM values which were more than 20 in one group while less than 20 in the other group) (Figure 5d). 5′‐tsRNAs (tRF‐5c and tiRNA‐5) subtype accounted for a significant proportion of tRFs and tiRNAs in both groups (Figure 5e). The 34 up‐regulated tRFs and tiRNAs could be grouped into 16 types based on the seed sequence (Table S2). Analysis of gene ontology terms suggested that the genes targeted by the 16 up‐regulated tRFs and tiRNAs are involved in neurogenesis and nervous system development (Figure 5f). The above results suggested that the changes of aging human sperm tsRNAs were related with nervous system development, which was similar with the results of mice.

## 3 DISCUSSION

Advanced paternal age has recently been recognized as a potential contributor to risk of psychiatric and neurological diseases in offspring (Janecka et al., 2017), but little is known about the mechanisms involved. In this study, we demonstrated that advanced paternal age in mice was associated with increased anxiety‐like behavior in F1 males, which was consistent with previous reports (Foldi et al., 2019; Krug et al., 2020). Those mice also exhibited deficits in social behavior, as demonstrated by increased preference for the empty chamber over the chamber containing a stranger mouse. A similar finding was reported in a mouse model of autism (Sampino et al., 2014). F1 mice that developed from zygotes that had been injected with sperm total RNAs or tsRNAs from aged mice also spent less time in the central area of the open field test and on the open arm of the elevated plus–maze test, but the number of entries into the open arm of the elevated plus‐maze test was significantly lower in the F1 mice that had been injected with sperm tsRNA from aged mice. We speculated that sperm tsRNA from aged fathers was mainly related to anxiety‐like behavior in F1 offspring and more tsRNA injection was associated with more severity. However, social abnormality has not been observed in F1 mice from injection of total sperm RNA, which suggested that sperm RNA might not involve in regulating the activity of social behavior in F1 offspring.

According to the length and cleavage sites, tsRNAs can be divided into tiRNAs, produced by specific cleavage in the anticodon loop of mature tRNAs; and tRFs, derived from primary or mature tRNAs (Li et al., 2018). We found that the 69 up‐regulated tRFs and tiRNAs consisted mainly of 5′‐tsRNAs (tRF‐5c and tiRNA‐5). Previous studies showed that 5′‐tsRNAs, but not 3′‐tsRNAs, can regulate translation by interfering with translation initiation or RNA modification, or by acting like miRNAs to repress translation (Shi et al., 2019). We hypothesize that age‐related changes in sperm tsRNA dysregulate gene expression in pre‐implantation embryo. In support of this idea, our KEGG analysis showed that the genes targeted by 69 up‐regulated tRFs and tiRNAs in Ma group were significantly enriched in pathways related to neural signal transduction and synaptic function.

Our transcriptome data indicate that tsRNAs injection in zygotes have downstream effects and resulted in reprogramed gene expression of the cerebral cortex and hippocampal tissues in F1 offspring. The DEGs including the genes encoding TH, TPH2, GABRR2, GABRA6, GABRA2, NGFR, and BDNF, which involved in dopaminergic synapses and neurotrophin signaling pathways. As reported before, the decreased expression of TH and TPH2, both rate‐limiting enzymes in dopamine synthesis, reduces levels of dopamine in the prefrontal cortex and hippocampus (Lu et al., 2019). Altered expression of Gabrr2, Gabra6 and Gabra2 is implicated in the pathogenesis of anxiety and depression (Bravo et al., 2011; Cryan & Kaupmann, 2005). BDNF is one of the most important regulators of brain signaling and synaptic plasticity (Kowianski et al., 2018), and the BDNF‐TRKB signaling pathway plays an important role in cognitive function, memory, and anxiety. Reduction in BDNF levels is associated with anxiety‐like behavior (Gibney et al., 2013). We hypothesize that tsRNAs in Ma sperm mediated abnormal expression of these genes may be responsible for the increased anxiety‐like behavior in F1 mice in the Ma group. tsRNAs injection in zygotes have dysregulate gene expression in pre‐implantation embryos, which echoes RNA‐seq of cerebral cortex and hippocampus in F1 mice. These transcriptional changes in pre‐implantation embryos may cause profound downstream effects in the brain of F1 offspring, which may give rise to anxiety‐like behaviors.

Consistent with our observations in mice, the different human sperm tRFs and tiRNAs in the aged and young men were mainly of the 5′ tsRNA subtype. A total of 358 tRFs and tiRNAs were expressed in humans and 384 in mice, of which 111 were common. These results indicate a relatively well conserved sperm tRF/tiRNA signature between the two species. An analysis of enrichment of gene ontology terms showed that the genes targeted by 34 up‐regulated tRFs and tiRNAs in aged men were mainly involved in neurogenesis and nervous system development. This suggests that tsRNAs target genes are important for nervous system development and behavioral traits.

Hua et al. reported that sperm tsRNA can be used as a marker to assess sperm quality and embryonic quality in IVF (Hua et al., 2019). Subsequently, Chen et al. reported human sperm tRNAGln‐TTG is highly associated with sperm quality (Chen et al., 2021). These results showed that sperm tsRNA may be a potential biomarker for the evaluation of male fertility. Similarly, our finding that tRF and tiRNA expression clearly differed between aged and young men implies that human sperm tsRNAs may be useful as biomarkers of aging‐related diseases.

Nonetheless, there are some limitations. Firstly, we injected zygotes with tsRNA mixtures rather than single tsRNA. Since sperm tsRNA adopt spatial structure and undergo various modifications. tsRNA synthesized *in vitro* may not behave identically to tsRNAs produced *in vivo* (Chen et al., 2016). Secondly, we only considered the men age and fertility as modifying factors and did not take into account some other factors (e.g., environmental pollution, lifestyle *etc*.). Thirdly, only F1 male mice were examined in the present study. The female reproductive cycle has an unpredictable effect on behavioral tests and more epidemiological and animal studies have reported a higher rate of neuropsychiatric disorders in males.

## 4 MATERIALS AND METHODS

### 4.1 Animals and sperm collection

Male and female C57BL/6N mice were purchased from the Shanghai Model Organisms Center (Shanghai, China). Animals were housed in cages at a temperature of 23±1°C and 45–60% relative humidity, with food and water ad libitum. Mice 14 to 18 months old were included in the aged male mice (Ma) group, while mice aged 3–4 months old were considered young male mice (My). Six male mice in each group were naturally mated with 10‐week‐old female mice in a ratio of 1:2. The female mouse was individually housed after pregnant. Only male progeny was selected at a maximum of two F1 mice for one F0 male mice and a total of 10 F1 male mice in each group were randomly selected for behavioral testing. All animal experiments complied with the regulations of the Animal Care and Use Committee of Fudan University and Tong ji University.

Sperm was isolated from the cauda epididymis of mice and processed for RNA extraction. The detailed method can be referred to our published study (Guo et al., 2020).

### 4.2 Human sperm collection

Human sperm was donated by men who were visiting the assisted reproduction center as part of *in vitro* fertilization procedures. All experiments were approved by the Ethics Committee of Shanghai First Maternity and Infant Hospital. All patients provided written informed consent. Men 45–50 years old were included in the aged male (Ha) group, while those between 25–27 years old were included in the young male (Hy) group. Sperm samples were obtained from both group after 3–5 days of sexual abstinence. Sperm quality parameters, including concentration, motility, and morphology, were evaluated according to World Health Organization guidelines (5th edition, 2010) to ensure that samples were normal. In addition, semen samples that led to clinical pregnancy during *in vitro* fertilization procedures (*n* = 3 for each group) were random selected for tsRNA‐seq.

### 4.3 Sperm RNA extraction and tsRNA isolation

In mice, sperm was collected into 3 ml of G‐IVF fertilization medium (Vitrolife, Sweden) and maintained for 20 min at 37°C in cell incubator containing 5% carbon dioxide. Then the supernatant was centrifugated to obtain sperm precipitate. Then the precipitate was treated with 1 ml of somatic cell lysis buffer (0.1% SDS, 0.5% Triton X100 in PBS). After washing with G‐IVF medium and centrifugation, 1 ml TRIzol reagent (Invitrogen) and 3–4 RNAase‐free grinding beads were added to the sperm precipitates for lysis in a homogenizer. Next, sperm RNA was extracted using the RNA Extraction Kit (Qiagen, USA) according to the manufacturer's protocol. Total sperm RNAs were separated by denatured 15% PAGE with 8 M urea, and RNAs of 28–40 nt (predominantly tsRNAs) were isolated and purified. Four replicate were set for the aged and young groups.

In human, the sperm precipitate was obtained from semen by density gradient centrifugation. The human sperm RNA extraction method was the same as described above in mice. Three replicate were set for the aged and young groups.

### 4.4 Library preparation, sequencing and Bioinformatics analysis of sperm small RNAs

Small RNA libraries were constructed using the NEBNext^®^ Multiplex Small RNA Library Prep Set (Illumina). Agarose electrophoresis was used to check the integrity of total RNA samples, whose concentration was quantified using a NanoDrop ND‐1000 instrument. Total RNA samples were first pretreated to remove RNA modifications that could interfere with small RNA‐seq library construction: 3’‐aminoacyl (charged) groups were deacetylated to 3’‐OH for 3’ adaptor ligation, 3’‐(2’,3’)‐cyclic phosphate groups were removed to leave 3’‐OH for 3’ adaptor ligation, 5’‐OH groups were phosphorylated to 5’‐P for 5’‐adaptor ligation, and m1A and m3C demethylation were removed for efficient reverse transcription. The completed libraries were quantified using a Agilent 2100 Bioanalyzer.

Libraries were mixed in equal amounts according to the quantification results, then sequenced using the NextSeq 500/550 V2 kit (#FC‐404–2005, Illumina) according to the manufacturer's instructions. Unqualified samples were excluded before data preprocessing.

Image analysis and base calling were performed using Solexa Pipeline (Off‐Line Base Caller software, version 1.8). Sequencing quality was examined using FastQC. The trimmed reads were aligned to the mature tRNA sequences, and only up to one mismatch was allowed. Reads that did not map were aligned using Bowtie software to precursor tRNA sequences, and again only one mismatch was allowed. The expression levels of tRFs and tiRNAs were calculated based on counts of mapped reads. Differentially expressed tRFs and tiRNAs were identified based on counts, using the *edgeR* package in R.

All steps in the library construction, sequencing and bioinformatics analysis of small RNA were performed by KangChen Bio‐tech (Shanghai, China).

### 4.5 Procedures with mouse zygotes

Female C57BL/6N mice (8 weeks old) were induced to superovulate with 5 IU pregnant mare serum gonadotropin followed 48 h later with 5 IU human chorionic gonadotropin, then mated with C57BL/6N male mice (10 weeks old). Zygotes were collected from the oviducts, and eight to ten picoliters total sperm RNA or the fraction enriched for tsRNA (concentration, 2 ng/μl) were injected into the cytoplasm of 8–10 zygotes at a time (Chen et al., 2016). Zygotes were cultured until the two‐cell embryo stage, then transferred into the fallopian tube of pseudopregnant mice or prepared for single‐cell RNA‐seq.

### 4.6 RNA‐seq of pre‐implantation embryos

RNA‐seq libraries were constructed as described (Liu et al., 2016) using three independent samples of two‐cell embryos or blastocysts from the Ma and My groups. In brief, two‐cell embryos or blastocysts were removed polar body and transferred into the lysis buffer for reverse transcription. A poly(A) tail was added to the 3′ end of the first‐strand cDNA using terminal deoxynucleotidyl transferase. The total cDNA library was amplified for 18–20 cycles, then used for RNA‐seq library construction. Paired‐end 150‐bp sequencing was performed using a HiSeq X10 (Illumina, USA) at Berry Genomics Corporation (Beijing, China). The library was functionally annotated using the Database for Annotation, Visualization and Integrated Discovery resource. Genes with adjusted *p* < 0.05 and fold‐change (FC) ≥1.5 were considered DEGs. KEGG pathway enrichment analysis was performed (http://www.genome.jp/kegg).

### 4.7 Mouse behavioral assays

All the behavioral assay apparatus and software were purchased from Shanghai Xin Ruan Technology (Shanghai, China). Supermaze software was used to automatically track and record mouse tracks and generate data files for further analysis. At least 10 F1 male mice from each group were tested at the age of 10–12 weeks. Mice were allowed to acclimate in the behavior assessment room for at least 1 hour before testing. All behavior assessments took place at a light level of 40–50 lux in the test room. Each apparatus was cleaned with 75% ethanol at the end of each test in order to avoid odor interference on the next mouse.

For open field test, the open field consisted of a black box (50 cm ×50 cm ×50 cm) divided equally into nine squares. The central square was defined as the central area. A video camera was placed above the box to track mice movement. The mouse was placed in the open field and allowed to freely explore the arena for 5 min. After habituation, the total distance travelled and the time in the central area were recorded during 10 min. A reduction in time spent in the central area was interpreted as an increase in anxiety‐like behavior. The total distance travelled was used to assess locomotor activity. An entry was defined as the movement of all four paws into the central area.

For elevated plus‐maze test, the plus‐shaped maze consisted of two open and two closed arms, all elevated 100 cm above the ground. The two closed arms were 35 × 5 cm with 15 cm high walls, while the two open arms did not contain walls. A video camera was placed above the apparatus to track mouse movement. The mouse was placed in the center of the maze and tracked for 5 min. An entry was defined as the movement of all four paws into an arm. Time spent in open and closed arms and numbers of entries into open and closed arms were recorded in order to assess anxiety‐related behavior. Fewer entries or shorter time spent in the open arms were indicative of increased anxiety‐like behavior.

For three‐chambered social interaction test, the three‐chambered apparatus consisted of a transparent box (60 × 40 ×22 cm) divided into three chambers and containing two openings, which allowed the mouse to move freely among the three chambers. The left and right chambers contained wire mesh cups that held a stranger mouse or were empty. A video camera was placed above the chamber to track mouse movement. The subject mouse was placed in the central chamber for 10‐min habituation. A clean empty wire mesh cup (novel object) was placed in one side chamber, while an adult male conspecific mouse that had had no previous contact with the subject mouse (stranger mouse) was placed in a wire mesh cup in the other side chamber. Two doors between the three chambers were raised to begin the 10‐min sociability test. Time spent by the subject mouse sniffing within 2 cm of the wire cup containing the unfamiliar mouse or the novel object were recorded by the software in order to assess sociability.

For marble‐burying test, the mouse was placed in an open box (50 ×50 ×50 cm) containing fresh sawdust (approximately 5 cm deep) with 20 clean marbles prearranged in a 5 × 4 grid. The mouse was allowed to bury the marbles for 10 min to test repetitive stereotypic behavior. Marbles were considered buried if they were at least 2/3 covered with sawdust.

### 4.8 Isolation of mouse cerebral cortex and hippocampus

F1 male mice (three per group) were deeply anesthetized with isoflurane, then the brain was removed from the calvarium. The cerebral cortex and hippocampus were isolated and one part was immediately snap‐frozen in liquid nitrogen, while the other part was placed into RNAlater (Qiagen, USA) for RNA extraction.

### 4.9 RNA‐seq of mouse brain tissue

One mL TRIzol reagent (Invitrogen) and 3–4 RNAase‐free grinding beads were added to the cerebral cortex and hippocampus samples for lysis in the homogenizer. RNA was extracted using an RNA Extraction Kit (Qiagen, USA) according to the manufacturer's protocol. RNA quality was examined by gel electrophoresis and using a Nanodrop spectrophotometer (Thermo, Waltham, MA, USA). Oligo (Brandt et al., 2019)‐attached magnetic beads were used to purified mRNA. Purified mRNA was fragmented into small pieces with fragment buffer at appropriate temperature. Then First‐strand cDNA was generated using random hexamer‐primed reverse transcription, followed by a second‐strand cDNA synthesis. Afterward, A‐Tailing Mix and RNA Index Adapters were added by incubating to end repair. The cDNA fragments obtained from previous step were amplified by PCR, and products were purified by Ampure XP Beads, then dissolved in EB solution. The product was validated on the Agilent Technologies 2100 bioanalyzer for quality control. The double stranded PCR products from previous step were heated denatured and circularized by the splint oligo sequence to get the final library. The single‐strand circle DNA (sscir DNA) was formatted as the final library. The final library was amplified with phi29 to make DNA nanoball (DNB) which had more than 300 copies of one molecular, DNBs were loaded into the patterned nanoarray and single end 50 bases reads were generated on BGIseq500 platform (BGI‐Shenzhen, China). The sequencing data was filtered with SOAPnuke. Afterward, clean reads were obtained and stored in FASTQ format. The clean reads were mapped to the reference genome using HISAT2. Bowtie2 was applied to align the clean reads to the reference coding gene set, then expression level of gene was calculated by RSEM. The thresholds for determining DEGs were *p* < 0.05 and FC ≥1.5. Then the DEGs were chosen for function and signaling pathway enrichment analysis using the Gene Ontology and KEGG databases. Pathways were defined as significantly enriched when they contained at least two DEGs and when *p* < 0.05. The library preparation and sequencing analysis were performed by BGI (Shenzhen, China).

### 4.10 qPCR of genes in the cerebral cortex and hippocampus of F1 mice

The cDNA of cerebral cortex and hippocampus was synthesized using 5x all in one rt master mix (Abm, G490, Canada). Levels of mRNAs encoding BDNF, TH, Gabra2, and Gapdh (as internal control) were analyzed using Chamq Universal SYBR qPCR Master Mix (Vazyme, Q711‐02, China) and the ABI 7500 Real‐Time PCR System. Levels of mRNAs were normalized to those of the internal control. All the experiments were performed in biological triplicate, each in technical triplicate for both groups. The primers used for qPCR are shown in Table 3.

### 4.11 Statistical analysis

Data were presented as mean ±standard error of mean (SEM). We performed the D'Agostino–Pearson normality test and all experimental groups were passed the normal test. Two‐way comparisons were assessed for significance using a two‐tailed Student's t test, with *p* < 0.05 considered significant. All data were plotted and analyzed using Prism 7.0 software (GraphPad Software, La Jolla, CA, USA).

## CONFLICTS OF INTEREST

Yi Guo, Dandan Bai, Wenqiang Liu, Yingdong Liu, Yalin Zhang, Xiaochen Kou, Jiayu Chen, Hong Wang, Xiaoming Teng, Ji Zuo, Shaorong Gao declare that they have no conflict of interest. All procedures followed were in accordance with the ethical standards of the responsible committee on human experimentation (Shanghai First Maternity and Infant Hospital, Shanghai, China) and with the Helsinki Declaration of 1975, as revised in 2000. Informed consent was obtained from all patients for being included in the study. All institutional and national guidelines for the care and use of laboratory animals were followed.

## AUTHOR CONTRIBUTIONS

XT, JZ and SG designed the experiments. YG, DB and WL performed the most experiments and analyzed the data. YL, YZ, XK, JC and HW contributed reagents and helped with experiments. YG, DB, WL and SG wrote the manuscript.

## Supporting information

Supplementary Material

## Data Availability

All original sequence datasets have been submitted to the database of the National Center for Biotechnology Information (NCBI) Sequence Read Archive under accession number PRJNA685274 for mouse data and PRJNA684424 for human data.
