# Supplementary material for: Altered sperm tsRNAs in aged male contribute to anxiety‐like behavior in offspring
Source: Aging Cell. 2021 Aug 27;20(9):e13466. doi: 10.1111/acel.13466 (PMC8441364; doi:10.1111/acel.13466)
Supplement: Supplementary file 1 — Supplementary Material [file ACEL-20-e13466-s001.pdf]

Supplementary data

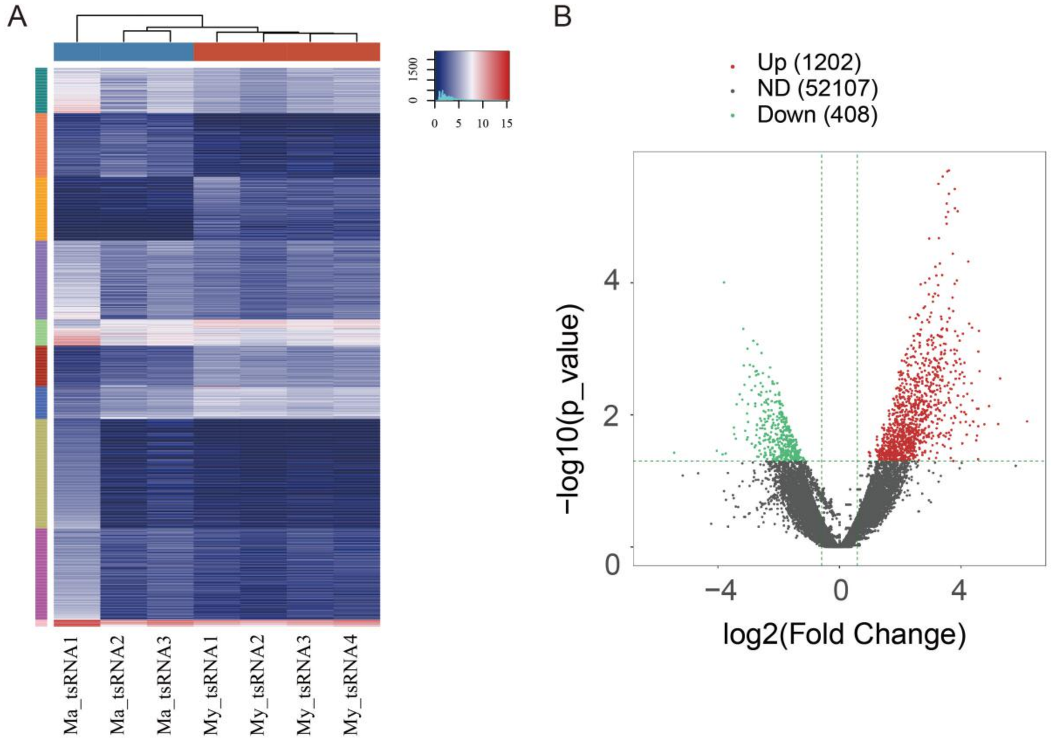

Fig. S1. Differences in sperm tsRNAs between aged male mice (Ma, n = 3) and young male mice (My, n = 4). (A) Heat map of sperm tsRNAs differentially expressed between the Ma group and My group. (B) Volcano plot of differentially expressed tsRNAs. tsRSA, transfer RNA-derived small RNA; Ma, aged male mice; My, young male mcie.

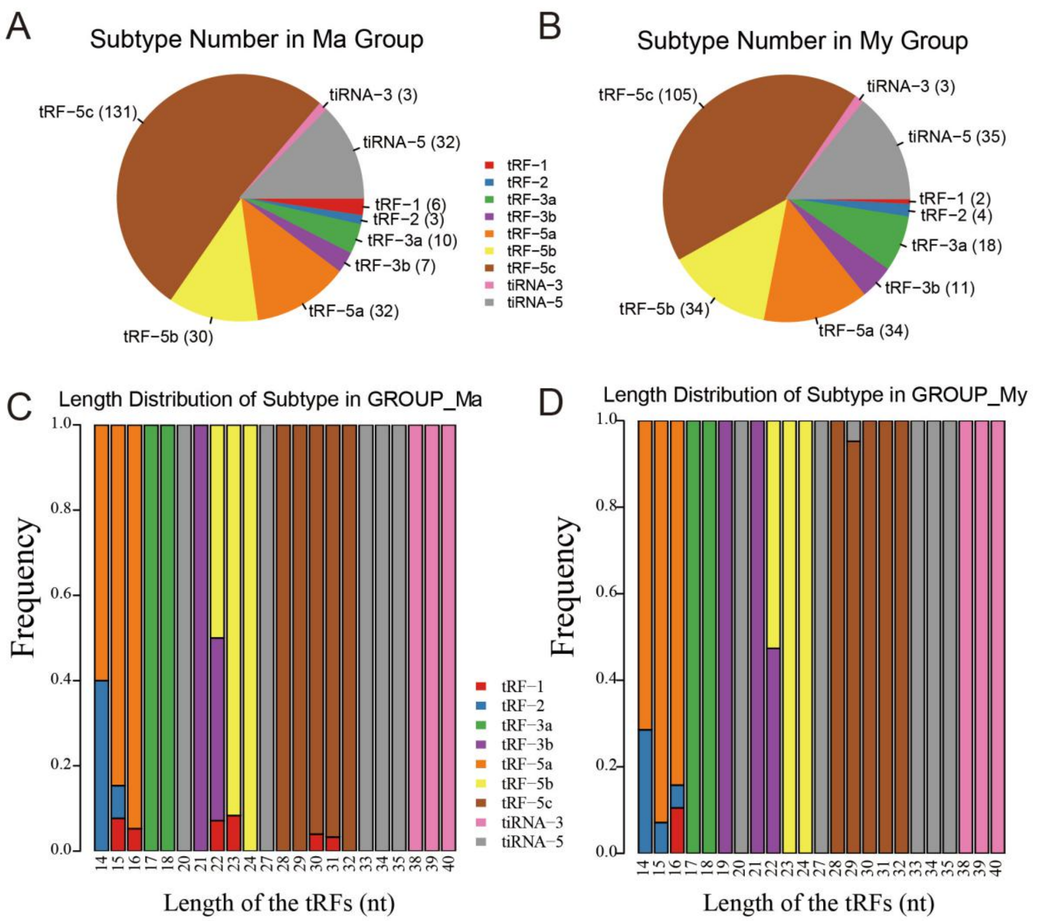

Fig. S2. Distribution of tRNA-derived fragments (tRFs and tiRNAs) across subtypes in the (A) Ma group and (B) My group. (C-D) The distribution of subtypes as a function of length. Ma, aged male mice; My, young male mice.

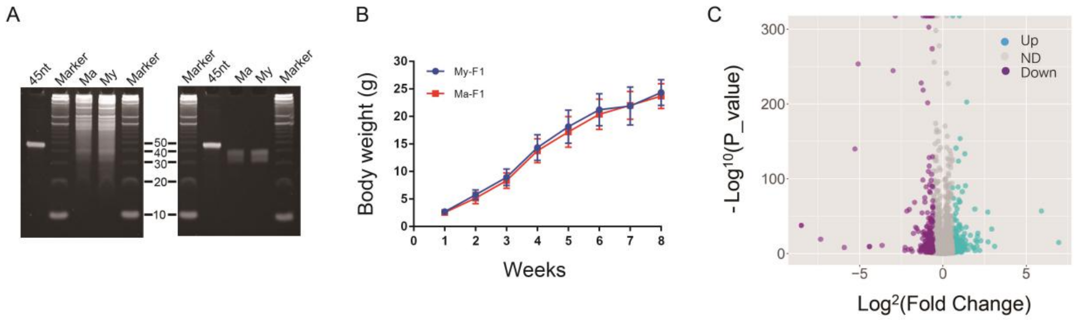

Fig. S3 Additional results obtained from F1 male mice arising from zygotes injected with sperm tsRNA from aged or young mice. (A) PAGE image of isolated sperm tsRNA fragments. (B) Growth curves of F1 males. (C) Volcano plot of genes differentially expressed between the Ma and My groups in the cerebral cortex and hippocampal tissues. tsRSA, transfer RNA-derived small RNA; Ma, aged male mice; My, young male mice.

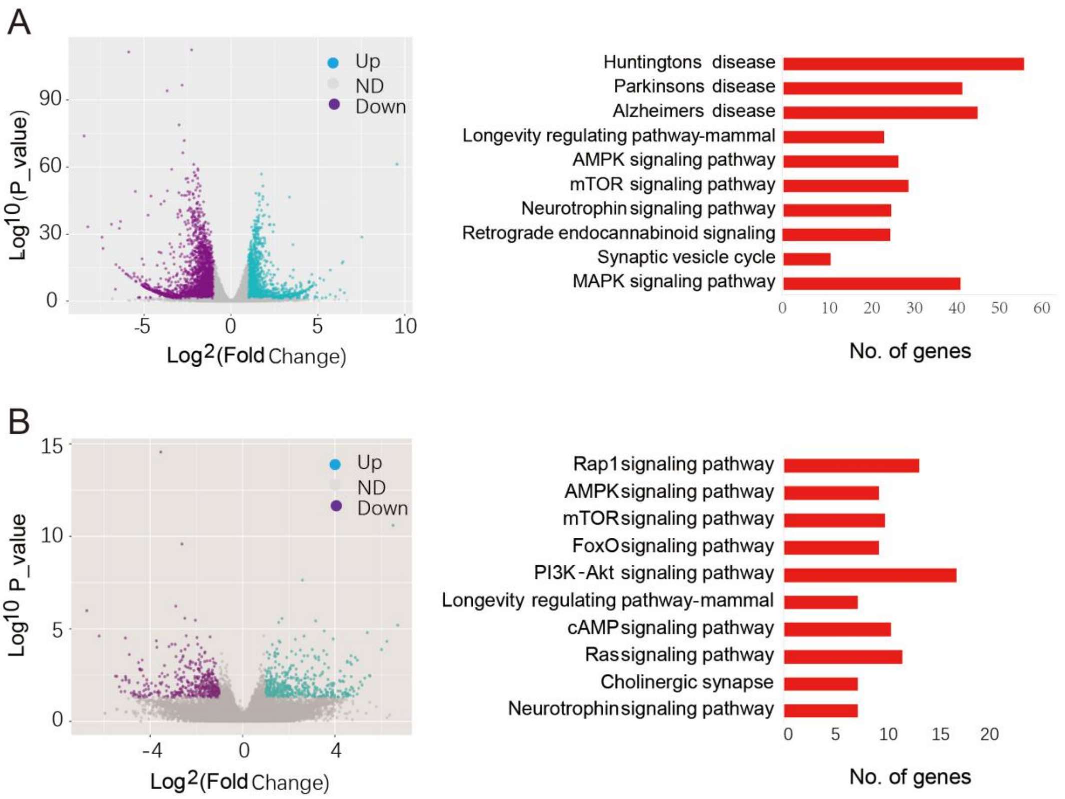

Fig. S4. Additional results of blastocysts and two-cell embryos after injection with sperm tsRNA from aged or young mice.

(A) Volcano plot of significantly differentially expressed genes in blastocysts between aged mice (Ma) and young mice (My). KEGG pathway analysis of genes up- or down-regulated in blastocysts of the Ma group. (B) Volcano plot of significantly differentially expressed genes in two-cells between aged mice (Ma) and young mice (My). KEGG pathway analysis of genes up- or down-regulated in two-cells of the Ma group. Data are from n = 3 independent samples for each group. tsRSA, transfer RNA-derived small RNA; Ma, aged male mice; My, young male mice.

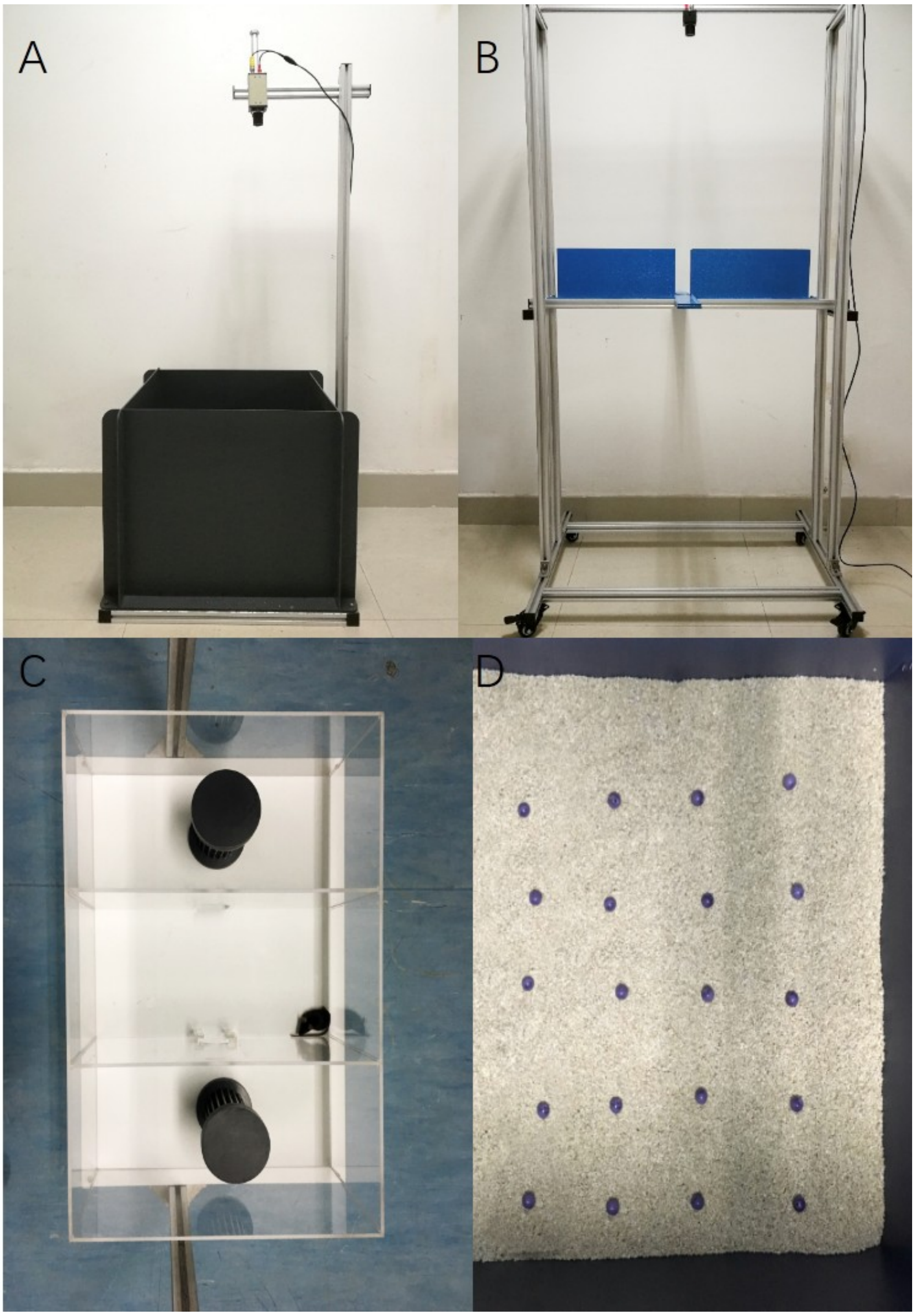

Fig. S5. Instrumentation used in behavioral assays. (A) Open field test. (B) Elevated plus maze test. (C) Three-chambered social interaction test. (D) Marble burying test.

Table S1. Sperm tRNA-derived fragments (tRFs) and tRNA-derived stress-induced RNAs (tiRNAs) up-regulated in aged male mice (n = 22)

ID	Sequence	Type	Length (nt)
tiRNA-Gly-TCC-003	GCGTTGGTGGTATAGTGGTGAGC ATAGCTGCCTT	tiRNA-5	34
tRF-Ala-CGC-023	TCCCTGGTAGTCTAGTGGTTAGG ATTCGGT	tRF-5c	30
tRF-Gln-CTG-018	GGTTCCATGGTGTAATGGTTAGC ACTCTGG	tRF-5c	30
tRF-Gln-TTG-014	GGTCCCATGGTGTAATGGTTAGC ACTCTGGA	tRF-5c	31
tRF-Glu-CTC-017	TCCCTGGTGGTCTAGTGGTTAGG ATTTGG	tRF-5c	29
tRF-Glu-TTC-028	TCCCATATGGTCTAGCGGTTAGG ATTCCTGG	tRF-5c	31
tRF-Glu-TTC-022	TCCCACATGGTCTAGCGGTTAGG ATTCCTGG	tRF-5c	31
tRF-Gly-CCC-008	GCATTGGTAGTTCAATGGTAGAA TTCTCGC	tRF-5c	30
tRF-Gly-CCC-014	GCATTGGTGGTTCAATGGTAGAA TTCTCG	tRF-5c	29
tRF-Gly-CCC-033	GCGCCGCTGGTGTAGTGGTATCA TGCAAG	tRF-5c	29
tRF-Gly-GCC-009	GCATGGGTGGTTCAGTGGTAGAA TTCTC	tRF-5c	28
tRF-Gly-GCC-019	GCATTGGTGGTTCAGTGGTAGAA TTCTTG	tRF-5c	29
tRF-Gly-TCC-024	GCGTTGGTGGTATAGTGGTGAGC ATAGCTGC	tRF-5c	31
tRF-His-GTG-016	GCCGAGATCGTATAGTGGTTAGT ACTCTGCA	tRF-5c	31
tRF-His-GTG-023	GCCGTGATCGTATAGTGGTTAGT ACTCTGCG	tRF-5c	31
tRF-Lys-TTT-020	GCCCGGATAGCTCAGTCGGTAGA GCATCAGA	tRF-5c	31
tRF-Pro-AGG-011	GGCTCGTTGGTCTAGGGGTATGA TTCTCGC	tRF-5c	30
tRF-Val-AAC-016	GTTTCCGTAGTGTAGTGGTTATCA CATT	tRF-5c	28
tRF-Val-AAC-030	GTTTCTGTAGTGTAGTGGTTATCA CGCTCGC	tRF-5c	31

tRF-Val-TAC-019	GGTTCCATAGTGTAGCGGTTATC ACGTCTG	tRF-5c	30
tRF-Val-CAC-013	GTTTCCCTAGTGTAGTGGTTATCA CGTTCGC	tRF-5c	31
tRF-Val-CAC-016	GTTTCCGTAGTGTAGTGGTTATCA CGCTC	tRF-5c	29

Table S2. Sperm tRNA-derived fragments (tRFs) and tRNA-derived stress-induced RNAs (tiRNAs) up-regulated in aged men (n = 16)

ID	Sequence	Type	Length (nt)
tRF-Leu-TAA-029	ATTCCTCTTCTTAACACCA	tRF-3b	19
tRF-Leu-AAG-001	ATCCCACCGCTGCCACCA	tRF-3a	18
tRF-Leu-TAA-030	CAATTCCTCTTCTTAACACCA	tRF-3b	21
tRF-Ser-TGA-004	GAAAAAGTCATGGAGGCCATG GGGTTGGCT	tRF-5c	30
tRF-Ser-TGA-005	GAAGCGGGTGCTCTTAT	tRF-1	17
tRF-Ser-GCT-025	GACGAGGTGGCCGAGTGGTTAA	tRF-5b	22
tRF-Ser-TGA-012	GCAGCGATGGCCGAGTGGTTAAGG	tRF-5b	24
tRF-Pro-AGG-019	GGCTCGTTGGTCTAGGGGTATG	tRF-5b	22
tRF-Ala-AGC-008	GGGGATGTAGCTCAGTGGTAGA GCGCATGCTT	tRF-5c	32
tiRNA-Leu-TAG-004	GGTAGTGTGGCCGAGCGGTCTA AGGCGCTGGATTT	tiRNA-5	35
tRF-Ser-AGA-013	GTAGTCGTGGCCGAGTGGTTAA	tRF-5b	22
tRF-Leu-TAA-037	GTTAAGATGGCAGAGCCCGGTA	tRF-5b	22
tRF-Phe-GAA-023	GTTTATGTAGCTTACCTCCTCAA	tRF-5b	23
tRF-Glu-TTC-009	TCCCACATGGTCTAGCGGTTAGGA TTCC	tRF-5c	28
tRF-Ala-AGC-010	TCCCCGGCACCTCCACCA	tRF-3a	18
tRF-Arg-CCT-001	TCGAGAGGGGCTGTGCTCGCAAGGTTTC TTT	tRF-1	31

Table S3. Sequence of primers used for quantitative polymerase chain reaction.

	Sequence	
**Gene**	**Sense 5'-3'**	**Antisense 5'-3'**
BDNF	TTACCTGGATGCCGCAAACAT	TGACCCACTCGCTAATACTGTC
TH	TTGGCTGACCGCACATTT	GCCCCCAGAGATGCAAGT
Gabra2	GGACCCAGTCAGGTTGGTG	TCCTGGTCTAAGCCGATTATCAT

BDNF, Brain derived neurotrophic factor; Gabra2, Gamma-aminobutyric acid receptor subunit alpha-2; TH, Tyrosine hydroxylase.

Table S4. The results of natural mating

	Ma	My
male mice	6	6
mated female	12	12
F1 generation(male)	43	49
F1 generation(female)	37	34

Ma, aged male mice; My, young male mice

Table S5. The statistical results of zygotes injection of RNA

	Group	No. of injected zygotes	No. of transferred 2-cell embryos	No. of Surrogate females	Live born (%transfer)
Sperm total RNA injection	Ma	132	120	8	32 (26.7%)
	My	125	113	8	30 (26.5%)
Sperm tsRNA injection	Ma	302	284	15	61 (21.4%)
	My	252	231	15	51 (22.1%)

Ma, aged male mice; My, young male mice

Table S6. Sperm parameters

Sample ID	sperm concentration (10^6/mL)	sperm volumn (μl)	RNA concentration (ng/μl)	RNA Volume (μl)	RNA Quantity (ng)
My 1	15.2	500	32.85	15	492.75
My 2	18.2	500	36.26	15	543.9
My 3	21.7	500	40.08	15	601.2

My 4	23.1	500	40.51	15	607.65
My 5	16.4	500	36.03	15	540.45
Ma 1	13.5	500	30.46	15	456.9
Ma 2	21.8	500	26.78	15	401.7
Ma 3	13.8	500	27.35	15	410.25
Ma 4	19.4	500	41.99	15	629.85
Ma5	13.7	500	30.11	15	451.65

Ma, aged male mice; My, young male mice
